# The grain yield regulator *NOG1* plays a dual role in latitudinal adaptation and cold tolerance during rice domestication

**DOI:** 10.3389/fgene.2022.1039677

**Published:** 2022-11-11

**Authors:** Xing Huo, Junyi Xiao, Xin Peng, Yanhui Lin, Dilin Liu, Wuge Liu, Yilong Liao, Jinhua Li, Manshan Zhu, Chongyun Fu, Xueqin Zeng, Xiaozhi Ma, Le Kong, Feng Wang

**Affiliations:** ^1^ Guangdong Key Laboratory of New Technology in Rice Breeding, Guangdong Rice Engineering Laboratory, Rice Research Institute, Guangdong Academy of Agricultural Sciences, Guangzhou, China; ^2^ College of Agriculture, South China Agricultural University, Guangzhou, China; ^3^ Hainan Scientific Research Station of Crop Gene Resource and Germplasm Enhancement, Ministry of Agriculture, Hainan Key Laboratory of Crop Genetics and Breeding, Institute of Food Crops, Hainan Academy of Agricultural Sciences, Haikou, China

**Keywords:** *NOG1*, cold stress, domestication, differentially expressed genes, RNA-seq

## Abstract

Rice originated in tropical and subtropical regions and is distributed worldwide. Low temperature is one of the most critical abiotic stresses affecting grain yield and geographical distribution of rice. It is vital to elucidate the molecular mechanism of chilling tolerance in rice for ensuring cereals production. Previously we isolated the domestication-related gene *NOG1* which affects rice grain number and yield. In this study, we specified that rice varieties harboring high-yielding *NOG1* allele are more distributed in low-latitude regions. Additionally, we observed *NOG1* influences the chilling tolerance of rice. Through genome-wide transcriptional analysis after cold treatment at 10°C, there were 717 differentially expressed genes (DEGs) in *nog1* near-isogenic lines compared with the control Guichao 2, including 432 up-regulated DEGs and 284 down-regulated DEGs. Gene ontology annotations and KEGG enrichment analysis of DEGs showed that various biological processes and signaling pathways were related to cold stress, such as lipid metabolism and genetic information processing. These results provide new insights into the mechanism of chilling tolerance in rice and the molecular basis of environmental adaptation during rice domestication.

## Introduction

Rice is one of the most important food crops, feeding more than half of the world’s population (Ito and Lacerda, 2019). Coping with various biotic and abiotic stresses and maintaining stable yields during rice production is of great significance to ensuring world food security (Lesk et al., 2016). Low temperature is an essential abiotic stress factor affecting the environmental adaptability and geographical distribution of rice. Since rice originated in tropical and subtropical regions, it is extremely sensitive to low temperature. The damage caused by low temperature will reduce the growth and development of rice, and eventually lead to the reduction of yield and quality (Jaglo et al., 2001; Zhang et al., 2019). Cold stress is prevalent in the world’s main rice producing areas, and leads to huge economic losses every year. There are more than 15 million hectares of rice in the world facing the threat of low temperature (Lin et al., 2004; Zhang et al., 2014; Pradhan et al., 2019; Li et al., 2021). As an essential abiotic stress, cold stress has been a primary spot in plant science research. A series of cold tolerance-related genes were described (Saito et al., 2004; Fujino et al., 2008; Fujino and Matsuda, 2010; Su et al., 2010; Liu et al., 2013; Ma et al., 2015; Manishankar and Kudla, 2015; Shi and Gong, 2015; Mao et al., 2019). However, the molecular mechanisms of cold stress tolerance in rice are still unclear. In recent years, RNA-Seq has been widely used to perform genome-wide gene expression analysis, providing detailed and in-depth data for the molecular mechanism study of plant cold tolerance (Bashir et al., 2019; Wai et al., 2021). *NOG1* is a functional gene that regulates grain number and yield in rice. The functional site of *NOG1* is a 12-bp copy number variation in the promoter region. There are two haplotypes of *NOG1* in cultivated rice, with two copies of 12-bp fragments in high-yielding rice and only one copy of 12-bp in low-yielding rice (Huo et al., 2017). In this study, we found that the yield-related *NOG1* is associated with the geographic distribution of rice varieties and acts as a negative player in cold tolerance during rice domestication. The genome-wide transcriptional identification of *NOG1* near-isogenic lines under cold stress were additionally compared, which provided new insights into the molecular mechanism of *NOG1*-mediated cold tolerance pathway in rice.

## Results

### Geographical distribution pattern of *NOG1* alleles

**FIGURE 1:**
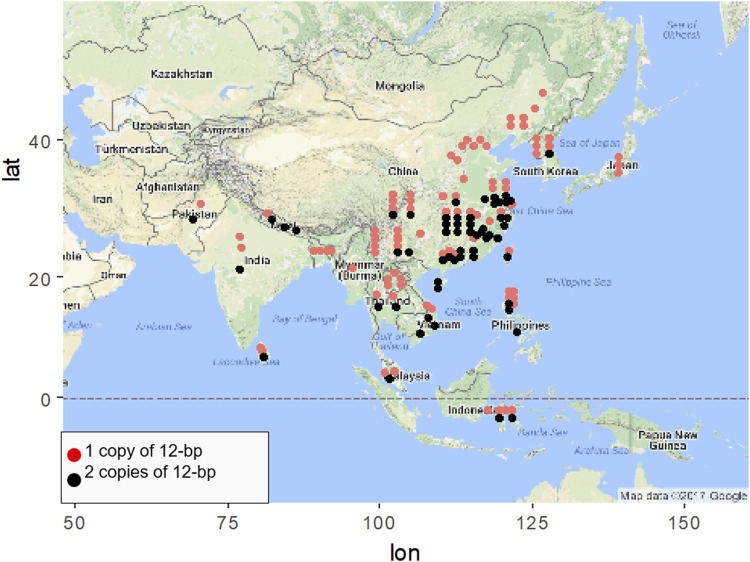
Geographical distribution of rice varieties containing the diverse *NOG1* alleles.

Geographic distribution of different *NOG1* alleles were investigated using 158 rice accessions, including 84 *indica* and 74 *japonica* varieties (Supplementary Tables S1,S2). As a result, high-yielding rice varieties containing two copies of 12-bp fragment were more frequently distributed in low-latitude regions (Figure 1). Under the control of other variables, the relationship between latitude and temperature is inversely proportional, that is, the higher the latitude, the lower the temperature. The principle is that the altitude of the Sun decreases with increasing latitude, resulting in a decrease in solar radiation with increasing latitude, resulting in a decrease in temperature with increasing latitude. The geographic distribution of *NOG1* alleles suggests that *NOG1* might affect the response to cold stress in addition to its role in improving rice yield.

### Construction of *NOG1* near-isogenic line

**FIGURE 2:**
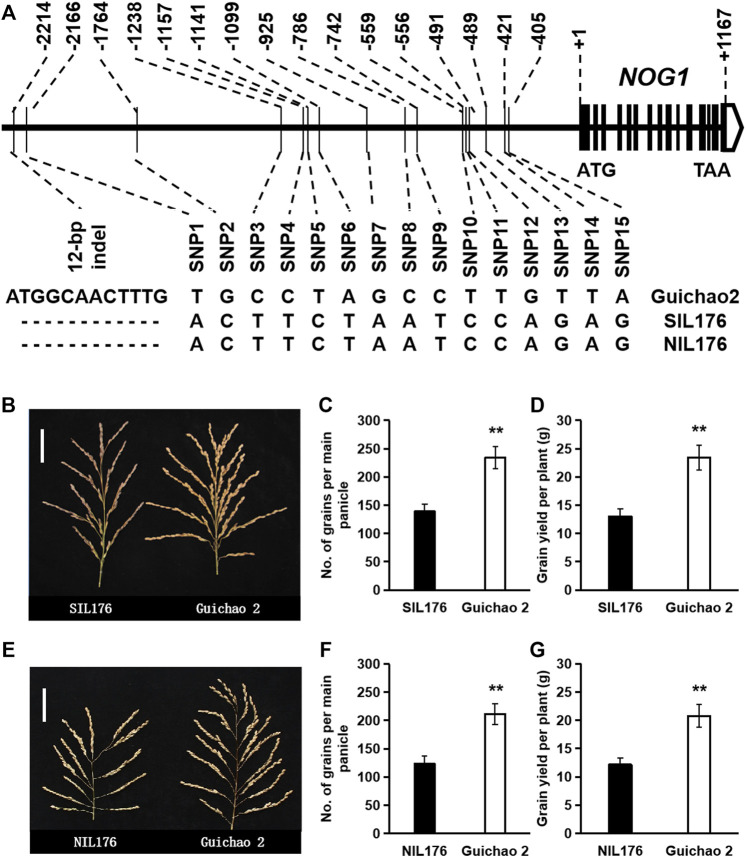
Genotypes and phenotypes of the *nog1* near-isogenic line. **(A)**: Genotypes of the *nog1* near-isogenic line (NIL176), SIL176 and Guichao 2. **(B–D)**: Phenotypic comparison between SIL176 and Guichao 2. **(E–G)**: Phenotypic comparison between NIL176 and Guichao 2.

In order to further eliminate the interference of genetic background, SIL176, an introgression line harboring *nog1* allele in the background of Guichao 2, was selected to backcross with Guichao 2 and then selfed. Combined with genotype identification in the BC_2_F_2_ population, a near-isogenic line with only one fragment introgressed in the genomic region of *nog1* compared with Guichao 2 was screened and named NIL176. Gene sequencing validated that the *NOG1* sequence in NIL176 was identical to that in SIL176. Compared with Guichao 2, NIL176 has a 12-bp deletion and 15 SNPs in the promoter region (Figure 2A), the grain number per panicle and grain yield of the near-isogenic lines were investigated, and the results showed that NIL176 exhibited a similar performance as SIL176. Compared with Guichao 2, both NIL176 and SIL176 exhibited less grains per panicle and lower yield (Figures 2B–G). Therefore, in the further study of the function of *NOG1* gene and its response to cold stress, using near-isogenic lines as materials can eliminate the interference of genetic background and obtain more reliable results.

### Altered resistance of *NOG1* near-isogenic line to cold stress

**FIGURE 3:**
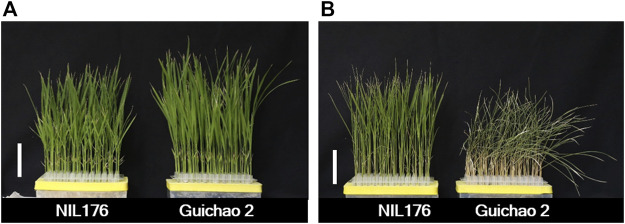
Phenotypes of chilling tolerance between NIL176 and Guichao 2. **(A)**: Phenotypic comparison of NIL176 and Guichao 2 at 28°C. **(B)**: Phenotypic comparison of NIL176 and Guichao 2 after cold stress.

To verify whether *NOG1* affects the response of rice to cold stress, NIL176 and Guichao 2 were used for further study. Rice seedlings were treated under low temperature stress at 10°C for 10 days and recovered at 28°C for three days to examine the phenotype. After cold stress, NIL176 still maintained a normal growth, while Guichao 2 showed obvious cold injury phenotypes including wilting and yellowing (Figure 3). The results showed that compared with Guichao 2, the tolerance of NIL176 to cold stress was significantly improved.

### RNA sequencing and differentially expressed genes in response to cold stress

**FIGURE 4:**
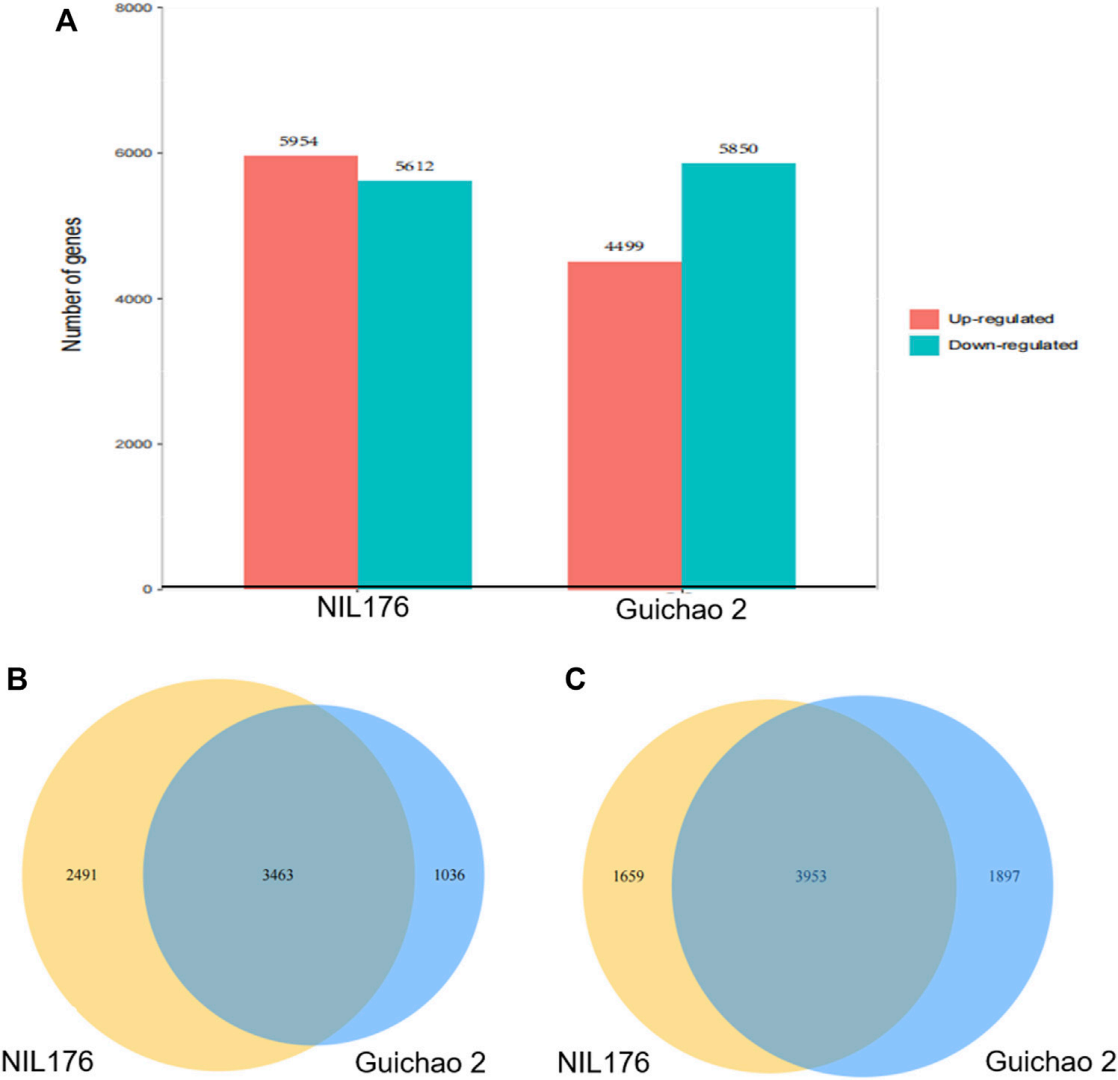
Comparisons of differentially expressed genes between NIL176 and Guichao 2 under cold stress. **(A)**: Numbers of differentially expressed genes in NIL176 and Guichao 2. **(B)**: Up-regulated differentially expressed genes in NIL176 and Guichao 2. **(C)**: Down-regulated differentially expressed genes in NIL176 and Guichao 2.

RNA-seq analysis was performed for Guichao 2 and NIL176, which were subjected to low temperature treatment at 10°C for 12 h. The differentially expressed genes (DEGs) were screened with the difference of gene expression levels more than 2 folds or less than 0.5 fold. After cold treatment, NIL176 had 5,954 up-regulated DEGs and 5,612 down-regulated DEGs, while Guichao 2 had 4,499 up-regulated DEGs and 5,850 down-regulated DEGs (Figure 4A). NIL176 and Guichao 2 shared 3,463 up-regulated genes and 3,953 down-regulated genes (Figures 4B,C). Under cold stress, the number of down-regulated genes in NIL176 and Guichao 2 were highly close. However, the up-regulated genes in NIL176 were significantly more than those in Guichao 2. A total of 2,491 up-regulated genes were unique in NIL176.

### Functional classification of data and differentially expressed genes

**FIGURE 5:**
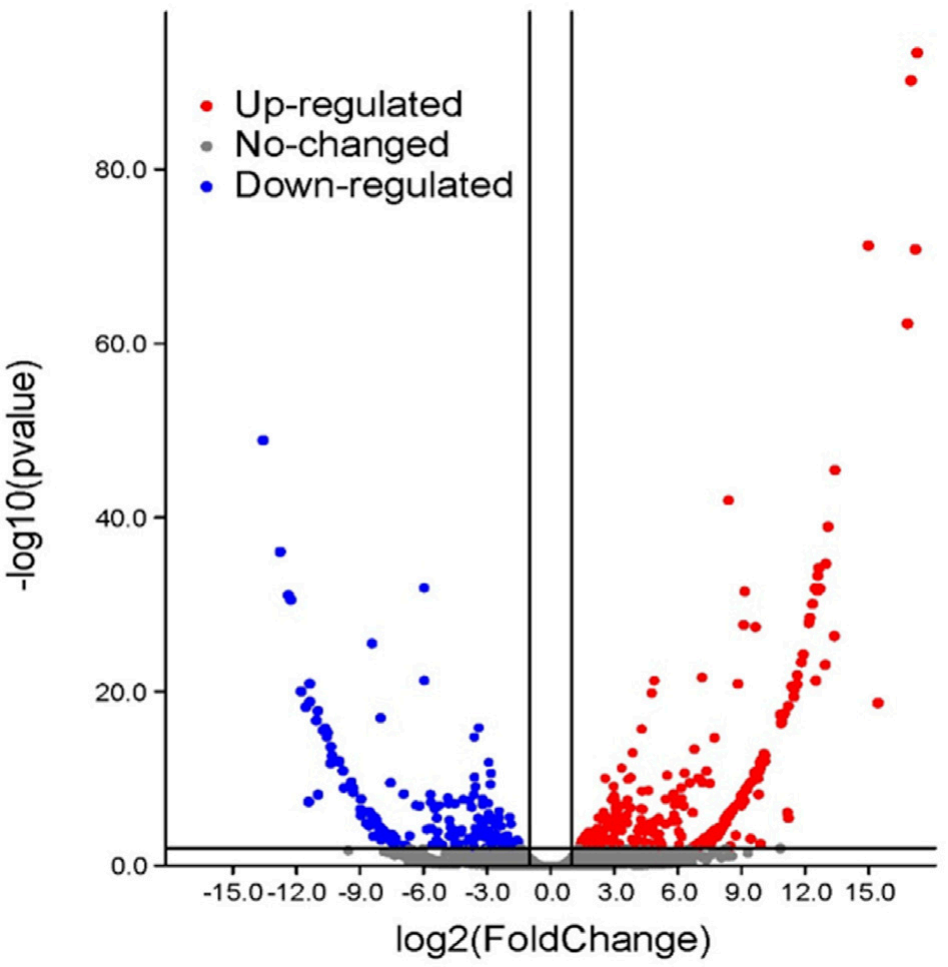
Volcano plot between NIL176 and Guichao 2. After cold-treatment, there are a total of 717 significantly differentially expressed genes in NIL176 compared with Guichao 2, including 432 up-regulated genes and 284 down-regulated genes.

**FIGURE 6:**
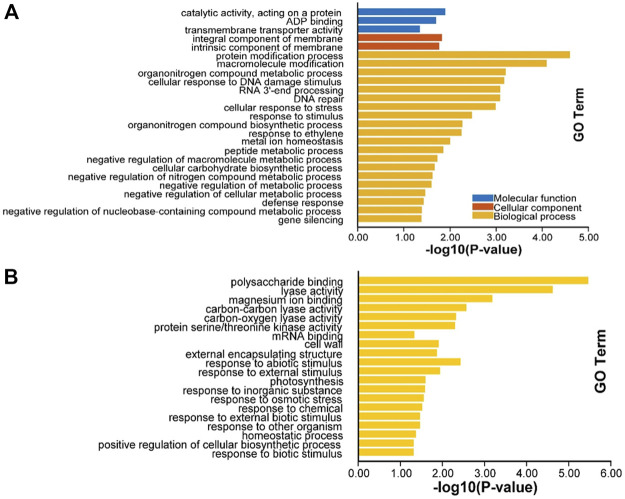
GO enrichment of differentially expressed genes between NIL176 and Guichao 2. **(A)**: GO enrichment of up-regulated genes in NIL176 compared with Guichao 2. **(B)**: GO enrichment of down-regulated genes in NIL176 compared with Guichao 2.

After cold treatment, there are 717 DEGs when compared NIL176 with Guichao 2, of which 432 were up-regulated and 285 were down-regulated (Figure 5 and Supplementary Table S3). In order to further analyze the molecular mechanism of *NOG1*-mediated response to cold stress in rice, Gene ontology (GO) annotation and KEGG pathway enrichment analysis were conducted to identify cold stress-related pathways using DEGs between NIL176 and Guichao 2. The up-regulated DEGs were involved in biological processes such as cellular response to stress, response to stimulus, and defense response. It also affects cellular components including integral component of membrane and intrinsic component of membrane (Figure 6A). Down-regulation DEGs involves biological processes such as external condensing structure, response to abiotic stimulus, and response to inorganic substance (Figure 6B).

**FIGURE 7:**
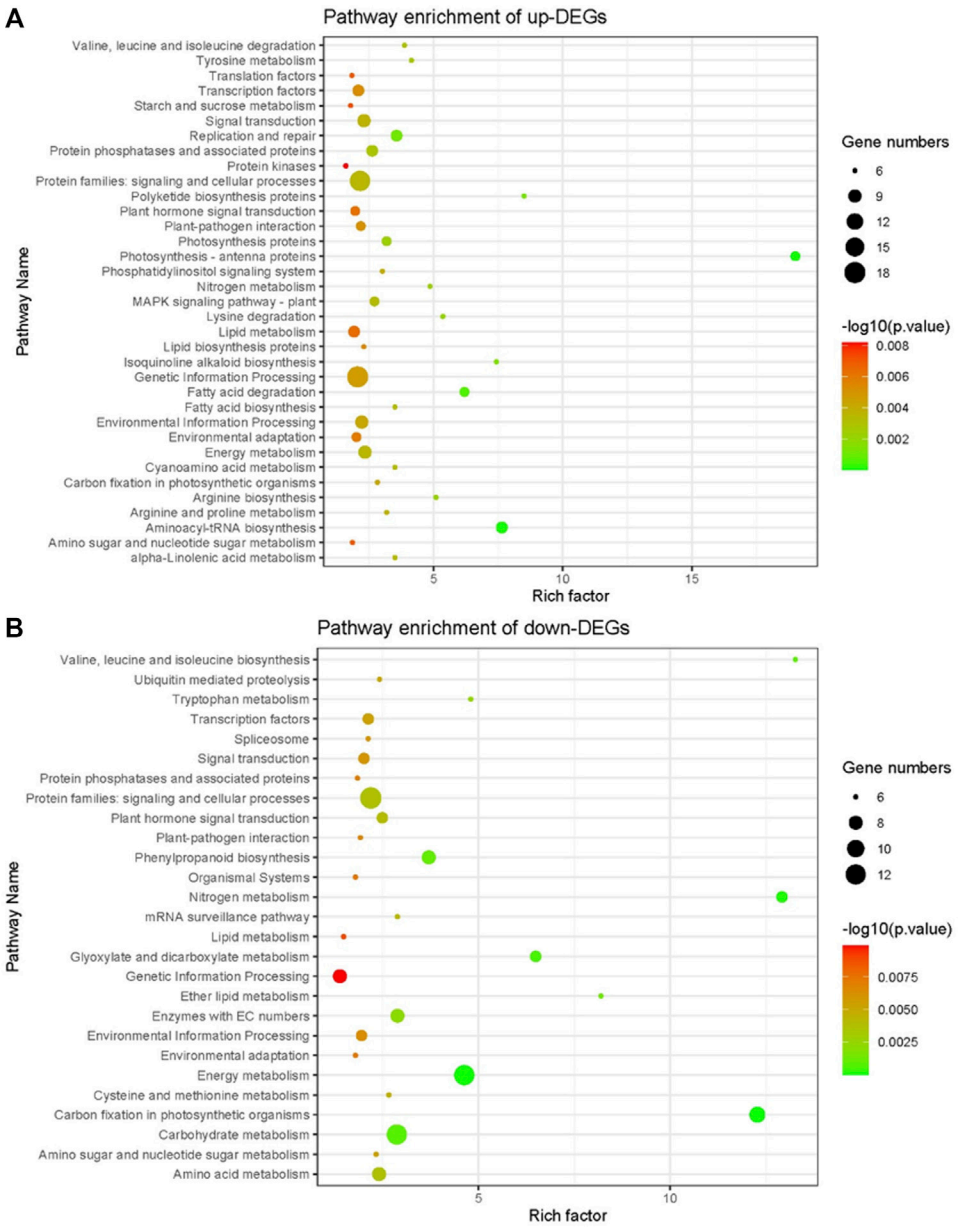
KEGG pathway enrichment of differentially expressed genes between NIL176 and Guichao 2. **(A)**: KEGG pathway enrichment of up-regulated genes in NIL176 compared with Guichao 2. **(B)**: KEGG pathway enrichment of down-regulated genes in NIL176 compared with Guichao 2.

KEGG enrichment analysis showed that up-regulated DEGs were enriched to lipid metabolism, genetic information processing, protein families: signaling and cellular processes, protein kinases and other pathways (Figure 7A), and down-regulated DEGs were enriched to genetic information processing, carbohydrate metabolism, energy metabolism, and other pathways (Figure 7B). Notably, pathways such as genetic information processing, lipid metabolism, protein families: signaling and cellular processes can be enriched in both up-regulated and down-regulated DEGs, indicating that these pathways may play an important role in the *NOG1*-mediated rice cold tolerance gene network.

### Fatty acid related gene involved in *NOG1*-mediated cold stress

**FIGURE 8:**
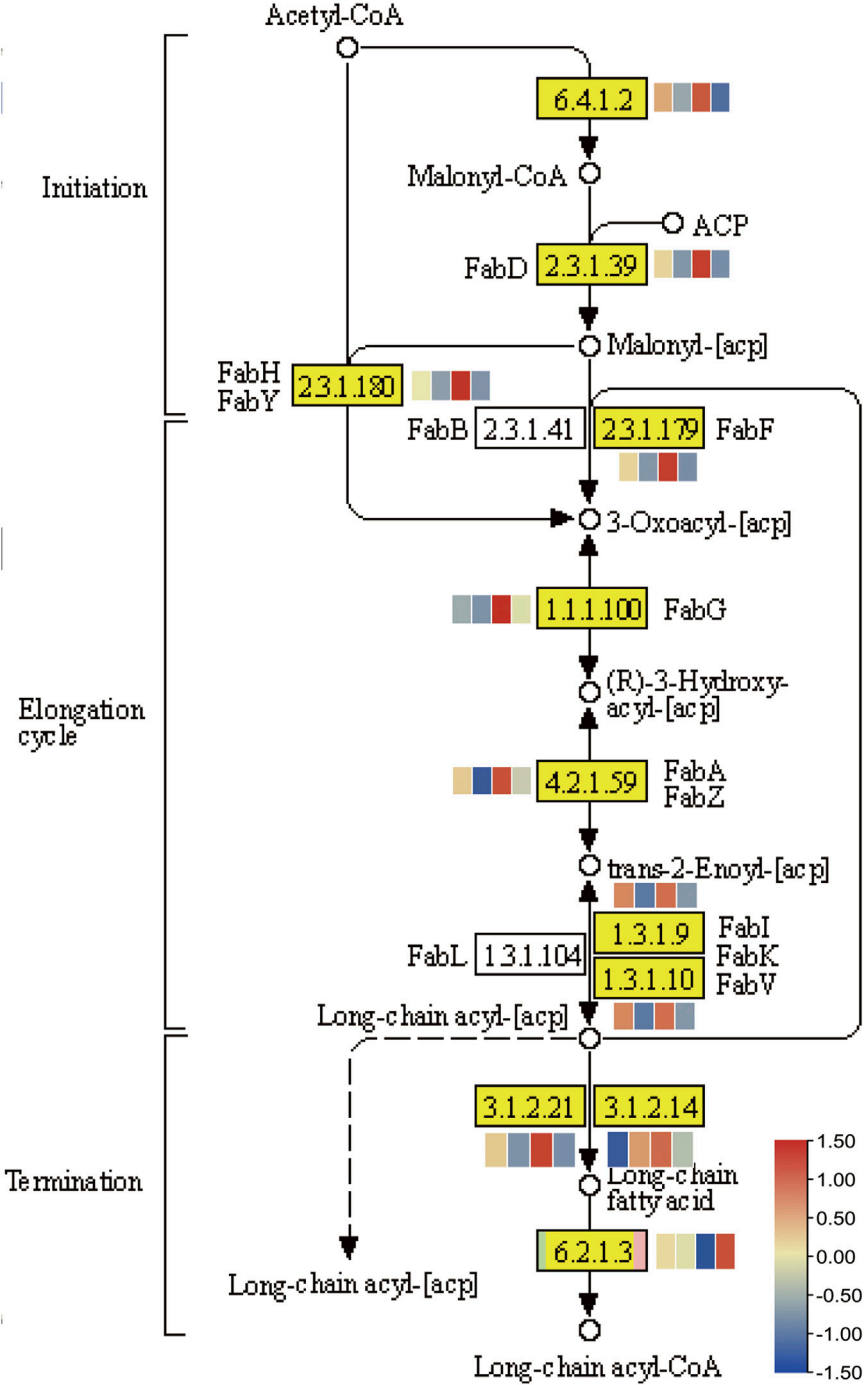
The KEGG pathway of fatty acid biosynthesis. The heatmap was generated by using the FPKM values of Guichao 2 under normal condition, Guichao 2 after cold treatment, NIL176 under normal condition, and NIL176 after cold treatment with row normalization.

Fatty acids, the major constituents of membrane glycerolipids, have important biological functions for stress response in plants. In response to a decrease in ambient temperature, plants increase the level of their unsaturated fatty acids, maintaining the appropriate fluidity of membrane lipids (Sakamoto and Murata, 2002). The related genes of fatty acid metabolism were enriched in DGEs of Guichao 2 and NIL176 (Figure 7). To investigate whether fatty acids involved in *NOG1*-mediated cold stress, we detected expression changes among fatty acids related genes (Figure 8). In the KEGG pathway of fatty acid biosynthesis, most of genes were down-regulated after cold treatment both in Guichao 2 and NIL176. However, seven genes are relatively high expression in NIL176 compared with Guichao 2 under normal condition, which suggests that NIL176 accumulates more fatty acids before cold stress, compared with Guichao 2. Long-chain acyl-CoA synthetase (LACS) plays a critical role in plant development and stress responses (Zhao et al., 2021). Intriguingly, the expression of *LACS* (K01897 [EC:6.2.1.3]) was particularly high expression in the cold-treated NIL176 plant.

## Discussion

### *NOG1* as a player in rice domestication and latitude adaptability

Rice is one of the earliest crops domesticated by humans. A series of key domestication traits were artificially selected, such as seed shattering, plant architecture, grain color and awn development (Konishi et al., 2006; Li et al., 2006; Sweeney et al., 2006; Tan et al., 2008; Zhu et al., 2011; Zhu et al., 2013; Hua et al., 2015). At the same time, rice originated in tropical and subtropical regions. During the process of domestication, local varieties need a series of adaptive improvements to the local environment, including photoperiod, temperature tolerance, drought and other stress resistance. In this study, the domestication-related *NOG1* gene affects the cold tolerance of rice, thereby affecting the latitudinal distribution of *NOG1* alleles. In the future molecular breeding process for genetic improvement using domesticated genes such as *NOG1*, it is necessary to comprehensively consider the geographic location and temperature of the planting area.

### Fatty acid metabolism pathway contributes to stress resistance in rice

Biofilm is the main damaged part of plants under cold stress, and the damage can lead to irreversible phase transition of the biofilm system. The fluidity and stability of biofilms are closely related to the low temperature tolerance of rice (Sakamoto and Murata, 2002). Fatty acids are important components of biofilms, and the content and composition of unsaturated fatty acids play an influential role in the cold tolerance of rice (Ariizumi et al., 2002). The *NOG1* gene that regulates grain number and yield in rice encodes enoyl-CoA hydratase/isomerase (ECH), a key enzyme in fatty acid metabolism, which affects the content of endogenous fatty acids and the composition of unsaturated fatty acids in rice, especially C18:3. The fatty acid biosynthesis pathway was enriched in DEGs, which indicates that fatty acid metabolism likely played an essential role in *NOG1*-mediated low temperature stress. Lipid metabolism, alpha-Linolenic acid metabolism, fatty acid biosynthesis and fatty acid degradation pathways were all enriched.

### Cold stress response pathways are involved in various biological processes

RNA-seq can analyze the differential expression of genome-wide genes, which is helpful to comprehensively analyze the response pathway of rice cold tolerance. The results of GO annotation and KEGG enrichment analysis of DEGs showed that in addition to fatty acid metabolism pathways, there are multiple pathways involved in cold stress responses. GO annotation enriched in transmembrane transporter activity, integral component of membrane, intrinsic component of membrane and response to inorganic substance. Membrane transporters are important functional proteins that change biomembrane permeability, maintain osmotic pressure and ion transport inside and outside the biomembrane, and are one of the essential ways to improve plant cold tolerance (Lissarre et al., 2010; Miura and Furumoto, 2013).

Additionally, cold stress is related to plant stress resistance (Saijo and Loo, 2020). In this study, GO analysis showed that stress resistance-related pathways such as external encapsulating structure, response to abiotic stimulus, and response to inorganic substance were activated. The results of KEGG also showed that environmental adaptation, environmental adaptation pathways such as Information processing and plant-pathogen interaction are enriched. Hence, *NOG1* may also be related to other abiotic stresses and disease resistance in rice in addition to its role in low temperature stress. These results enrich the understanding of cold resistance pathways in rice and provide new evidence for analyzing the molecular mechanism of rice cold tolerance.

## Conclusion

The *NOG1* gene, which regulates grain number and yield in rice, encodes a key enzyme in fatty acid metabolism. Allele identification and geographic origin of 158 rice varieties including 84 indica varieties and 74 japonica varieties indicated a strong latitude adaptation of the *NOG1* alleles. Through the cold treatment of Guichao 2 and NIL176 (the near-isogenic line of *nog1*) at the seedling stage, it was found that *NOG1* regulates the cold tolerance in rice. Using whole transcriptome analysis of near-isogenic lines, 5,954 up-regulated DEGs and 5,612 down-regulated DEGs were observed in NIL176, whereas 4,499 up-regulated DEGs and 5,850 down-regulated DEGs were found in Guichao 2 after cold treatment. The number of up-regulated genes in NIL176 was significantly higher than that in Guichao 2, among which 2,491 genes were only up-regulated in NIL176. GO annotation and KEGG enrichment analysis of DEGs showed that fatty acid metabolism, transmembrane transporter activity, integral component of membrane and plant-pathogen interaction pathways DEGs were enriched.

## Materials and methods

### Plant materials

The sources of SIL176 and Guichao 2 were introduced in previous studies (Huo et al., 2017). The near-isogenic line NIL176 was selected from BC_2_F_2_, using SIL176 as a donor and Guichao 2 as the recipient. A collection of 158 rice materials, including 84 indica (*O. sativa* L. ssp. *indica*) varieties and 74 japonica (*O. sativa* L. ssp. *japonica*) varieties, were used to verify the alleles of *NOG1* and to investigate the geographic distribution of material origins, as listed in Supplementary Table S1.

### Primers

The primers used in this study for NOG1 sequencing are listed in Supplementary Table S2.

### Cold stress identification at the seedling stage

The seeds of the test materials were treated at 45°C for 48 h to break dormancy. Soak seeds with ddH_2_0 at room temperature for 24 h, keep moist for 24–48 h to promote germination. Seeds with uniform whiteness were selected and sown in bottomless 96-well PCR plates. Plant 3 plates of each material, hydroponically in an artificial climate chamber. The temperature of the artificial climate chamber is 28°C, with a light/darkness regime of 12/12 h and a relative humidity of 85%. The tested materials were grown to the 3-leaf stage and subjected to low temperature treatment in the artificial climate chamber. The treatment temperature was 10°C, 12 h of light, 12 h of darkness, and a relative humidity of 85%. After 10 days of treatment, growth was recovered at 28°C for 3 days and the phenotype was investigated.

### RNA-sequencing and data analyses

Total RNA was extracted from the leaves of the plant with cold-treatment for 12 h using the QIAGEN plant RNA kit (Hilden, Germany). The concentration and quality of RNA were evaluated using NanoDrop 2000 UV-VIS spectrophotometer (NanoDrop Technologies, Wilmington, DE, United States). Paired-end reads were generated on a HiSeq2000 platform following the manufacturer’s instructions (Illumina, United States). The analysis of RNA-sequencing (RNA-seq) data and differentially expressed genes (DEGs) were carried out as previously reported (Peng et al., 2021). Briefly, RNA-seq data were mapped on the reference genome with HISAT2 2.1.0 (Kim et al., 2019). FeatureCounts 1.6.2 was used to count the number of reads mapped on exons (Liao et al., 2014). DEGs were evaluated by edgeR 3.32.0 (Robinson et al., 2010). Genes with *p* < 0.05 and log2 fold-changes > 1 were considered as DEGs. Further screening among the initial DEGs was performed based on fragments per kilo-base per million fragments mapped (FPKM) values. Afterward, the GO and KEGG annotations of DEGs were conducted by pannzer2 (Toronen et al., 2018) and KEGG Automatic Annotation Server (Moriya et al., 2007).

## Data Availability

The raw sequence data reported in this study have been deposited in the Genome Sequence Archive (Genomics, Proteomics & Bioinformatics 2021) in National Genomics Data Center (Nucleic Acids Res 2022), China National Center for Bioinformation/Beijing Institute of Genomics, Chinese Academy of Sciences (GSA: CRA008173) that are publicly accessible at https://ngdc.cncb.ac.cn/gsa (Chen et al., 2021).
